# A multi-objective scheduling optimization algorithm of a camera network for directional road network coverage

**DOI:** 10.1371/journal.pone.0206038

**Published:** 2018-10-31

**Authors:** Fei Gao, Meizhen Wang, Xuejun Liu, Ziran Wang

**Affiliations:** 1 Key Laboratory of Virtual Geographic Environment, Ministry of Education, Nanjing Normal University, Nanjing, China; 2 State Key Laboratory of Cultivation Base of Geographic Environment Evolution (Jiangsu Province), Nanjing, China; 3 Jiangsu Center for Collaborative Innovation in Geographical Information Resource Development and Application, Nanjing, China; 4 Taizhou College, Nanjing Normal University, Taizhou, China; Northeast Normal University, CHINA

## Abstract

Effective video monitoring systems require optimization of camera and road network coverage, to exploit fully the hardware and software solutions in smart city traffic applications. Monitoring requirements have grown increasingly diverse as scenes are becoming increasingly complex, thereby transforming the camera and road network coverage optimization issue into a nonlinear, high-dimension, and multi-objective problem. Previous research on this topic however, has focused on a single, specific optimization objective, which may result in invalid optimization results in actual applications. To extend this research, we propose a multi-objective scheduling optimization algorithm for a camera network that addresses the problem of directional road network coverage. In this solution, we incorporate an expanding parameter of main optical axes into particle swarm optimization algorithm. Our new strategy divides the range of main optical axes of all the cameras to control the scheduling number, achieving collaborative optimization of multiple objectives. In a simulated camera and road network, an experiment was designed for evaluating the effectiveness of the proposed method, comparing the distribution of optimization results with the global and local optimal solutions of the true value. A second experiment compared the distribution, performance and running time of the optimization results with different values of expanding parameter of main optical axes. A third experiment compared the performance of the optimization solutions with different values of camera parameters. The results showed that the proposed method can adapt to user application preference, and is effective and robust to schedule and allocate monitoring resources in different scenarios.

## Introduction

Monitoring cameras are sensors used to observe both static scenes and moving objects in the Internet of Things and Smart cities, given their real time, continuity, and reproducibility. In actual applications, cameras are typically deployed on the intersections of road network to monitor moving vehicles and pedestrians whose direction and trajectories are greatly constrained by the road network. These systems however, are not quite effective, as their low scheduling flexibility and cooperativity, which generates several challenges about how to improve coverage quality of available monitoring resources faced with various application demands. Therefore, the optimization problem of camera and road network coverage presents not only a key issue for video monitoring systems but also an interesting research direction for the academia.

The rest of the paper is organized as follows. Section 2 presents the related works about optimization problem of camera and road network coverage. Section 3 models the coverage optimization problem of camera on a directional road network. Section 4 presents our multi-objective scheduling optimization algorithm. Section 5 describes the experiment. Section 6 discusses the effectives of the proposed method, influence of the expanding parameter of main optical axes, and camera parameters. Section 7 concludes the paper.

## Related work

Solving the optimization problem of camera and road network coverage generally involves two key steps, modeling coverage optimization objectives and optimization processing [1, 2]. As the central issue in modeling, coverage optimization objectives have attracted the attention of many researchers who, in turn, have generated several interesting findings, and several optimization methods have been designed for such optimization objectives, especially the multiple-objective optimization which has good applicability in actual situations.

### Coverage optimization objectives

Several coverage optimization objectives have been proposed, such as strong barrier coverage[3], k-coverage [4], β-QoM coverage [5], and all-view coverage [6]. A strong barrier coverage can provide no-gap coverage that prevents intruders from passing through a region undetected, regardless of the crossing paths they choose. A camera network can provide k-barrier coverage for an ROI if at least k cameras can cover all the crossing paths throughout the region. β-QoM coverage focuses on the breadth of barrier coverage, which can guarantee high-quality monitoring along with the breadth of β, which in turn monitors the movement of intruders moving from one side of a squared region to the opposite side. A full-view covered object is always monitored by a camera regardless of its direction and when the viewing direction of camera is sufficiently close to the facing direction of the object. On this basis, several coverage optimization methods have been designed for different coverage optimization objectives.

### Coverage optimization methods

Some representative coverage optimization methods are voting strategy [7], method of geometry and graph theory [8, 9], greedy search [10], virtual potential field [11], and various heuristic algorithms, such as particle swarm optimization (PSO) algorithm [12], genetic algorithm (EA) [13], and simulated annealing algorithm [14]. Previous studies have established coverage optimization models that aim at specific optimization objectives and obtained excellent optimization results with different optimization methods. The aforementioned studies however, focus on a single, specific objective, while in actual applications, the optimization of camera and road network coverage is frequently constrained by many factors, which contribute to a typical multi-objective optimization problem. In this case, modeling a single optimization objective may not reflect monitoring quality comprehensively, thereby resulting in invalid optimization results. Therefore, to achieve a valid coverage optimization, a coverage model that comprises multiple optimization objectives must be designed.

### Multiple-objective optimization

As for the problem of multiple objective optimization, the processing methods can be generally categorized into two main types, 1) classical methods, such as objective weighting, distance functions and min-max formulation, 2) evolutionary methods, such as PSO, EA [15]. Classical methods, which convert a multi-objective problem into a single objective problem, are sensitive to weight vector and demand higher priori knowledge about the underlying problems, so the coordination among multiple objectives is greatly limited. In contrast, evolutionary methods, such as MOPSO, MOEA, have strong convergence, global optimization, and robustness, which contribute to the main optimization methods. What is more, plenty of effective improved strategies have been proposed, such as Pareto or tailored Pareto dominance strategy, where solutions having better non-dominance performance in the parent population are selected, some representative approaches are NSGA-II [16], MOPSO-CD[17], knee point driven evolutionary algorithm (KnEA) [18]. Decomposition strategy, where a complex multi-objective problem is decomposed into several single-objective problems or several simpler multi-objective problems, such as NSGA-III [19] and MOEA/DM [20]. Performance indicator strategy, where measurement quality of solutions is used as criteria of selection, and two widely used approaches are hypervolume-based evolutionary algorithm (HypE) [21] and an indicator based multi-objective evolutionary algorithm with reference point (AR-MOEA) [22]. Consequently, such processing methods and improved strategies, which adopt to sensing characteristics of cameras on a road network, can be introduced into the coverage optimization issue of camera network.

Therefore, we propose a multi-objective scheduling optimization algorithm for a camera network that addresses the problem of directional road network coverage, aimed to meet the various demands in actual applications and improve the effectiveness of the monitoring systems.

## Coverage optimization modeling of camera on a directional road network

In this paper, we focus on the coverage optimization problem of camera on a road network, therefore, it is necessary to build models of camera coverage and road network first. On this basis, we further define several concepts of valid coverage of camera on a directional road network, especially, expanding parameter of main optical axes. Finally, we conclude the most important aspects in common monitoring tasks, and design coverage optimization objectives.

### Camera coverage modeling

Monitoring cameras are generally classified into static cameras and pan-tilt-zoom (PTZ) cameras. The field of view (FOV) of a static camera is fixed if installed, while a PTZ camera has capability of dynamic monitoring and tracking with the adjustment of its focal length, rotating angle, and pitch angle despite the fixed FOV at some time. The actual FOV of a camera is a 3D truncated pyramid. To simplify the problem, we selected a sector model to represent the FOV of a camera in 2D.

**Fig 1:**
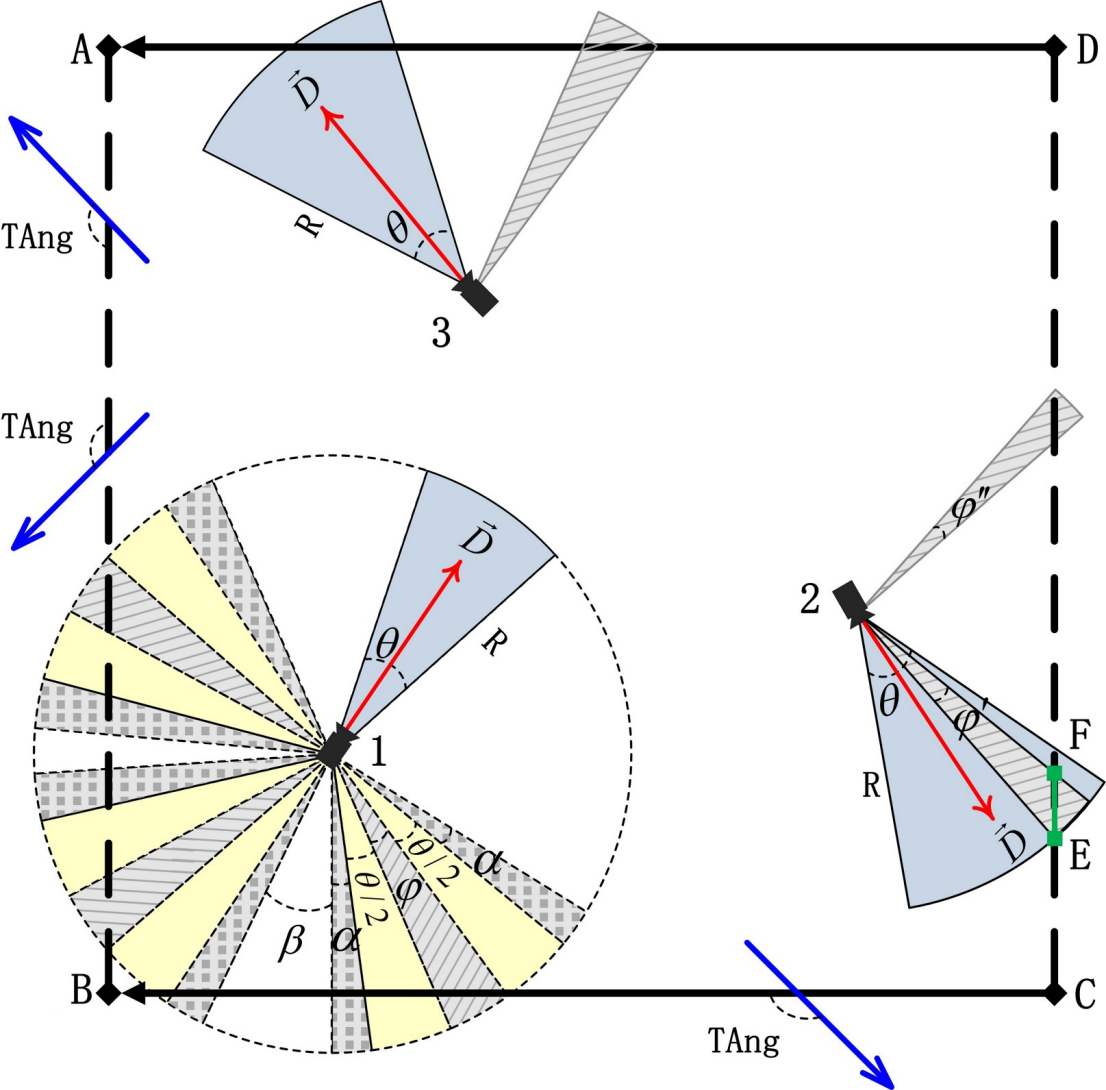
Valid coverage model of camera and directional road network.

The camera coverage model is denoted by a 4-tuple (P, $\vec{D}$, θ, R), where P is the spatial location (*x*, *y*) of a camera, and $\vec{D}$ denotes the current sensing direction of main optical axes (MOA). Unless otherwise specified, $\vec{D}=(dx,dy)$ denotes the unit length, where dx and dy are components along the x- and y-axes. Moreover, θ denotes the horizontal offset angle of FOV around $\vec{D}$, and R is the sensing range of a camera. Camera 1 is a PTZ camera, cameras 2 and 3 are static cameras, the blue sector represents FOV, and the red vector represents $\vec{D}$ (Fig 1).

Given that this paper focuses on the scheduling optimization of cameras instead of layout optimization, P remains unchanged and we assume that θ and R also remain unchanged after its initial deployment. As a result, the $\vec{D}$ of a static camera remains in its initial state all the time, while the range of $\vec{D}$ for PTZ cameras is denoted by the circular dotted line (Fig 1). Therefore, PTZ cameras can be dynamically scheduled by adjusting their $\vec{D}$ to change their coverage.

### Directional road network modeling

A road network comprises nodes and sections, and we use both of them to model. According to the constraints of advancing direction for moving objects, a road section can be divided into a directional or a bidirectional one. The former only permits unilateral movement from beginning to end, while the latter does not impose limits on the object along its advancing direction. A, B, C, and D are road nodes, D-A and C-B are directional road sections, while A-B and C-D are bidirectional road sections (Fig 1).

### Valid coverage modeling of camera on a directional road network

In actual situations, different monitoring tasks usually impose specific requirements for imaging angle. Under this constraint, not all the areas that can be monitored are considered as valid coverage areas. Furthermore, the topology of road network is relatively fixed, limiting the advancing direction and trajectories of moving objects. Therefore, valid coverage can be defined by the imaging angles of camera and road network. Given that not all the cameras need to be scheduled in a camera network scheduling solution, a method for dynamically controlling scheduling number of cameras must be devised. Based on the two aforementioned issues, we define the relevant concepts of valid coverage, and incorporate an expanding parameter of MOA. This new strategy divides the range of $\vec{D}$ of all the cameras to control the scheduling number.

#### Definition 1: Valid range of line of sight (LOS_V)

For a PTZ camera, LOS_V refers to the set of line of sight (LOS) vector that included angle with road section vector is no less than the threshold value (TAng), while the LOS_V of a static camera must be also in its initial FOV at the same time. Taking directional road section A-B and bidirectional road section C-D as examples (Fig 1), TAng represents the threshold value of included angle between the LOS of a camera and a road section vector, and φ represents the LOS_V of PTZ camera 1 on the directional road section C-B, shown as the sector field filled with slash. Two other LOS_Vs can be found on the bidirectional road section A-B. $\varphi^{\prime }$ represents the LOS_V of static camera 2 on the bidirectional road section C-D, and this is the sector field filled with slash within initial FOV of static camera 2. The LOS of φ″ satisfies the constraint of TAng but without initial FOV, therefore, it is not a LOS_V, shown as the sector field filled with slash without initial FOV of static camera 2. In the same way, static camera 3 does not have LOS_V on the directional road section D-A.

#### Definition 2: Valid range of main optical axes (MOA_V)

MOA_V represents the set of $\vec{D}$ corresponding to LOS_V. The yellow sector field represents half of θ. [φ-θ/2, φ+θ/2] represents the MOA_V of PTZ camera 1 on the directional road section C-B, while the MOA_V of static camera 2 on the bidirectional road section C-D remains its initial $\vec{D}$ (Fig 1).

#### Definition 3: Expanding range of main optical axes (MOA_E)

For a PTZ camera, MOA_E denotes the range of $\vec{D}$ whose upper and lower limits expand along with the length of MOA_V. The expanding parameter of MOA denoted by *k* represents the expanding multiples of the range of $\vec{D}$. α represents the MOA_E of PTZ camera 1 on the directional road section C-B where α = k*(φ+θ), and it is the gray sector field filled with dots (Fig 1). If $\vec{D}$∈MOA_V, the camera is selected to participate in the scheduling. On the contrary, if $\vec{D}$∈MOA_E, the camera is not selected to participate in the scheduling. In this way, we can dynamically control the probability for a PTZ camera to be selected by using expanding parameter of MOA. Therefore, the larger expanding parameter of MOA generates the larger MOA_E and eventually the smaller probability for a PTZ camera to be selected to participate in the scheduling. The static camera does not have MOA_E, if it has LOS_V, then this camera is surely selected, similar to the static camera 2. Otherwise, the static camera is surely not selected, similar to the static camera 3 (Fig 1).

#### Definition 4: Feasible range of main optical axes (MOA_F)

MOA_F refers to the union set of MOA_V and MOA_E, and it is the feasible solution space of $\vec{D}$. Those candidate cameras which participate in the scheduling must satisfy $\vec{D}$∈MOA_F. [φ-θ/2-α, φ+θ/2+α] represents the MOA_F of PTZ camera 1 on the directional road section C-B, while β represents one of its infeasible $\vec{D}$ sets, as shown the white sector field on the directional road section C-B (Fig 1).

#### Definition 5: Valid road section covered (Road_V)

The valid road section covered is the road section with LOS_V, the **valid length covered (Length_V)** is the length of covered part of **Road_V**, and the **valid camera (Camera_V)** is the camera whose $\vec{D}$∈MOA_V. $\varphi^{\prime }$ represents the LOS_V of static camera 2 on the bidirectional road section C-D and it is also the subset of initial FOV (Fig 1). Therefore, bidirectional road section C-D is a valid road section covered, segment E-F is the valid length covered, and static camera 2 is a valid camera. In the same way, static camera 3 is not a valid camera. While PTZ camera 1 cannot cover the target road network in its initial monitoring state, after adjusting $\vec{D}$, it can provide a valid coverage for directional road section C-B and bidirectional road section A-B. It should be emphasized that we only aim at valid coverage problems in this paper, they are valid camera, valid road section covered, and valid length of road network covered.

In conclusion, the coverage model of camera and directional road network can be denoted by a 7-tuple (P, $\vec{D}$, θ, R, Dis, TAng, *k*), where Dis denotes the shortest distance between a camera and a road network, and *k* denotes the expanding parameter of MOA. Given their fixed spatial location and topological relationship, Dis takes a constant value, while the values of TAng and *k* can be determined based on practical application requirements. In this case, the scheduling optimization solutions of camera and road network coverage only depend on the adjustment of $\vec{D}$.

### Optimization objectives modeling

As for common monitoring tasks, increasing length of road network covered can extend exposure time of monitoring objects, while increasing number of road sections covered can increase their capture probability. These two objectives are the most important aspects in monitoring quality. Given the limited monitoring resources and high economic cost, the coverage optimization of camera must focus on scheduling number of cameras. In this case, we choose the three objectives mentioned as the coverage optimization objectives by using Eqs (1)–(3) respectively.

$$Number_{\_road}(\{\vec{D}\})=\overset{n}{\underset{i=1}{\cup }}\left(Road\_V_{i}\right),\begin{array}{c} where \end{array}\begin{array}{c} \vec{D}\in \text{MOA}\_F \end{array}$$ (1)

$$Length_{\_road}(\{\vec{D}\})=\overset{n}{\underset{i=1}{\cup }}\left(Length\_V_{i}\right),\begin{array}{c} where \end{array}\begin{array}{c} \vec{D}\in \text{MOA}\_F \end{array}$$ (2)

$$Number_{\_camera}(\{\vec{D}\})=\sum_{i=1}^{n}(Camera\_V_{i}),\begin{array}{c} where \end{array}\begin{array}{c} \begin{array}{c} Camera\_V_{i}\text{=}1,if\begin{array}{c} \vec{D}\in \text{MOA}\_V \end{array}\\ Camera\_V_{i}\text{=}0,if\begin{array}{c} \vec{D}\in \text{MOA}\_E \end{array} \end{array} \end{array}$$ (3)

where $\left\{\vec{D}\right\}$ denotes the $\vec{D}$ set of a camera network, *n* denotes the number of cameras, $Number_{\_road}(\{\vec{D}\})$ denotes the number of road sections covered, $Length_{\_road}(\{\vec{D}\})$ denotes the length of road network covered, and $Number_{\_camera}(\{\vec{D}\})$ denotes the number of cameras participating in the scheduling. Therefore, the scheduling optimization problem of a camera network on a directional road network is a multi-objective optimization problem. This problem involves solving a set of scheduling optimization solutions $\left\{\vec{D}\right\}$ to increase the number of road sections covered, extends the length of road network covered and decreases the scheduling number of cameras, as shown in Eq (4).

$$F=f(Number_{\_road}(\{\vec{D}\}),Length_{\_road}(\{\vec{D}\}),Number_{\_camera}(\{\vec{D}\}))$$ (4)

$$Subject\;to:\{\begin{array}{c} \begin{array}{cc} Maximize & Number_{\_road}(\{\vec{D}\}) \end{array}\\ \begin{array}{cc} Maximize & Length_{\_road}(\{\vec{D}\}) \end{array}\\ \begin{array}{cc} Minimize & Number_{\_camera}(\{\vec{D}\}) \end{array} \end{array}$$

## A multi-objective scheduling optimization algorithm

We proposed a multi-objective scheduling optimization algorithm by incorporating an expanding parameter of MOA into PSO algorithm. In this case, the discrete variables, like scheduling number of cameras, will transform into continuous objectives, which generates a unify optimization strategy in PSO algorithm. The algorithm procedure is presented as follows (Table 1):

**Table 1:** A multi-objective scheduling optimization algorithm procedure of a camera network for directional road network coverage.

**Input: The camera network, directional road network and TAng**
**Output: The scheduling optimization solution set of camera network**
**Valid coverage computation:**
1. Initialize camera network and directional road network
2. Select cameras by distance
3. Compute LOS_V by TAng and select cameras by angle
4. Compute MOA_V
**Scheduling optimization:**
5. Compute expanding parameter of MOA
6. Compute MOA_E and MOA_F
7. Initialize particle population
8. Evaluate $P_{i}$ by using $Number_{\_road}(\{\vec{D}\})$, $Length_{\_road}(\{\vec{D}\})$, and $Number_{\_camera}(\{\vec{D}\})$
9. Repeat
10. Compute crowding distance value
11. Compute $P_{ibest}^{}$ and $P_{gbest}$
12. Update new velocity and position
13. Perform mutation
14. Perform constraint handing if $P_{i}^{}$ goes beyond MOA_F
15. Perform escape
16. Evaluate $P_{i}^{}$ by using $Number_{\_road}(\{\vec{D}\})$, $Length_{\_road}(\{\vec{D}\})$, and $Number_{\_camera}(\{\vec{D}\})$
17. Until the maximum number of generations or the rangeability of hypervolume is less than the threshold value.

The algorithm process consists of two steps, they are valid coverage computation, and scheduling optimization. The valid coverage computation step completes the initial modeling of camera and directional road network, selects the candidate cameras participating in scheduling, and obtains the MOA_V of cameras. In scheduling optimization, we expand the MOA_V to obtain both MOA_E and MOA_F by expanding parameter of MOA and solve the optimization objectives. Therefore, after inputting a camera network, directional road network, and TAng value into the proposed algorithm process, we determine the scheduling optimization solutions for the camera network.

### Valid coverage computation

The valid coverage computation is an important step before scheduling optimization. In this process, candidate cameras, which participate in scheduling can be selected under the constrains of distance and angle. We can also get MOA_V of cameras for later scheduling optimization.

#### Initialize the camera network and directional road network

Discretize the continuous target road network, begin the construction of nodes and sections, and initialize the $\left\{\vec{D}\right\}$ of cameras.

#### Select cameras by distance

Calculate distance between each camera and each road section. If the Dis is longer than the R, the camera has no abilities to cover the target road network. In this case, the camera will be deleted.

#### Compute LOS_V by TAng and select cameras by angle

According to the preset threshold value of included angle, calculate whether a camera has LOS_V on the road sections. If not, the camera will be deleted.

#### Compute MOA_V

Calculate the MOA_V based on the geometric relationship between $\vec{D}$ and LOS_V.

### Scheduling optimization

Scheduling optimization aims to schedule all the candidate cameras for better coverage, and expanding parameter of MOA plays an important role in this process. We describe its value range, important threshold values, influence mechanism on camera network scheduling, and compute its feasible range. Based on these works, PSO algorithm is applied to solve the scheduling optimization solutions. Furthermore, considering the sensing characteristics of cameras, we introduce several strategies to improve the applicability of PSO algorithm in this issue.

#### MOA_F computation

The feasible range of MOA is the valid flying space of particles in the optimization process, and it depends on the expanding parameter of MOA. In this section, we will compute the important threshold values of the parameter and further get MOA_F.

**Expanding parameter of MOA computation**. A camera network generally consists of multiple cameras, and each camera may have multiple MOA_Vs. For a MOA_V, an increase in expanding parameter of MOA will expand the range of MOA_E on both sides. In this case, the upper or lower limit of adjacent MOA_F will intersect and merge into a new interval. We call the value as the maximum *k* of the upper limit ($k_{U}$) or lower limit ($k_{L}$). With the continuous increasing of this parameter, all MOA_Fs will combine into one interval. At this point, the camera obtains a maximum of MOA_E and MOA_F, and we call this value as the maximum *k* of the camera ($k_{c}$). When the parameter increases to a certain value, all the cameras in the network obtain the maximum of MOA_F, and we call the value as the maximum *k* of the camera network ($k_{cn}$). The relationship among these three types of maximum *k* is computed as follows:

$$k_{Ui}=\frac{E_{Li+1}-E_{Ui}}{E_{Ui+1}-E_{Li+1}+E_{Ui}-E_{Li}}$$ (5)

$$k_{Li}=\frac{E_{Li}-E_{Ui-1}}{E_{Ui}-E_{Li}+E_{Ui-1}-E_{Li-1}}$$ (6)

$$k_{c}=maximize(k_{Ui},k_{Li}),i=1\cdots m$$ (7)

$$k_{c}{}_{n}=maximize(k_{j}),j=1\cdots n$$ (8)

where $E_{Ui}$ and $E_{Li}$ denote the upper and lower limits of MOA_V, $k_{Ui}$ and $k_{Li}$ denote the maximum expanding parameters of $E_{Ui}$ and $E_{Li}$, $k_{c}$ and $k_{cn}$ denote the maximum expanding parameters of a camera and camera network, and *m* as well as *n* denote the number of MOA_Vs and cameras.

**MOA_E and MOA_F computation**. According to the above analysis, after setting the expanding parameter of MOA, we can obtain MOA_E and MOA_F by expanding MOA_V.

#### PSO implementation

For the optimization problem of camera and road network coverage, each scheduling optimization solution is a particle whose dimension is denoted by the number of cameras. Considering that multiple-optimization generates non-dominated solution set, it is necessary to evaluate the performance of a single optimization solution as well as the optimization solution set. This section focus on these issues.

**Initialization of particle population**. We create a particle population by a preset solution scale, define MOA_Fs as the feasible solution space for each particle, set the maximum number of generations, and create an external archive to store the excellent particles.

**Performance evaluation of optimization solution**. We calculate objective function values of $Number_{\_road}(\{\vec{D}\})$, $Length_{\_road}(\{\vec{D}\})$, and $Number_{\_camera}(\{\vec{D}\})$ for each solution by using Eqs (1)–(3), respectively. As shown in Eq (4), if a solution is not worse than the others in these three optimization objectives, then this solution is called a non-dominated solution, and it must be stored in the external archive for the selection of global optimal solutions.

**Updating velocity and position**. To guide particles to fly within MOA_F, where not yet explored, and enhance the diversity of particle population, we use crowding distance [17] as an evaluation index to select the global optimal particles for updating velocity and position as follows:

$$V_{i}^{t+1}=\omega V_{i}^{t}+c_{1}r_{1}(P_{ibest}^{t}-P_{i}^{t})+c_{2}r_{2}(P_{gbest}-P_{i}^{t})$$ (9)

$$P_{i}^{t+1}=P_{i}^{t}+V_{i}^{t+1}$$ (10)

where $P_{ibest}^{}$ and $P_{gbest}$ denote the local optimum and global optimum, $V_{i}^{t}$ and $P_{i}^{t}$ denote the velocity and position of a particle, ω is the inertia weight, $c_{1}$ and $c_{2}$ are the learning factors, $r_{1}$and $r_{2}$ are random numbers generated between 0 and 1, and *t* is the current generation. $P_{ibest}^{}$ generates from the personal best states during the updating generations, while $P_{gbest}$ generates from the random selection of top portion in archive sorting in descending crowding distance values. In a way, maintenance strategy of external archive decides the optimization direction and distribution of particle population. All the new non-dominated solutions will be inserted into external archive after a new updating generation, meanwhile, all the solutions dominated by the new ones will be removed. If the archive is full, solutions will be randomly selected from bottom portion to make place for the new non-dominated ones. In this way, the particle population approaches the Pareto Front in trend during the updating generations.

**Performance evaluation of optimization solution set**. To quantitatively evaluate the comprehensive performance of optimization solution sets in different generations, we introduce the hypervolume metric [23], which refers to the size of the area governed by non-dominated solution set. It can comprehensively evaluate the convergence, uniformity, and universality of solution set [24]. Hypervolume is maximized if and only if a solution set only contains the Pareto Front [25]. Given the dimension differences, we need to normalize the three objective function values and then use the Hypervolume by Slicing Objectives (HSO) algorithm [26] to calculate hypervolume. In this case, we can obtain a more excellent scheduling optimization solution set.

#### Improved strategies

Given that the optimization problem of camera and road network coverage is a complex, high-dimensional problem, we introduce several improved strategies for better applicability of PSO algorithm in this issue, based on the sensing characteristics of cameras.

**Crowding distance computation**. Maintaining the diversity of particle population is critical in optimization process. To ensure a uniform distribution in the feasible solution space, crowding distance is introduced to measure the distribution density of the particles. The greater crowding distance corresponds to the sparser distribution and the greater diversity. In this case, the feasible solution space around the particles must be searched emphatically. The crowding distance is computed as follows:

$$P_{iCD}=\sum_{j=1}^{m}\left(S_{j}[i+1]-S_{j}[i-1\right])$$ (11)

Where $P_{iCD}$ is the crowding distance of $P_{i}$, *m* is the number of objectives, and $S_{j}[i]$ is the objective function value of $P_{i}$ order by objective *j*. We set *m* to 3 to denote the three objectives of $Number_{\_road}(\{\vec{D}\})$, $Length_{\_road}(\{\vec{D}\})$, and $Number_{\_camera}(\{\vec{D}\})$. Given the dimensional difference, we must normalize the three objective function values before calculating the crowding distance and then cumulate these values to obtain the crowding distance of each solution.

**Mutation**. To avoid premature convergence to the local optimal solution during the search process, we create a random disturbance to the obtained solutions for the diversity of particle population. We select cameras in these solutions randomly to make their $\vec{D}$ fluctuate in MOA_F.

**Constraint handling**. During its adjustment, $\vec{D}$ may fly out of MOA_F. In this case, constraint handling is performed to change flying direction of the particles to ensure a continued search process in MOA_F and obtain a feasible solution.

**Escape**. Given that a camera may have multiple independent MOA_Fs that may be far apart from one another, particles may have difficulties in flying toward other MOA_Fs by only following $P_{ibest}^{}$ or $P_{gbest}$. Therefore, we introduce the escape strategy [27] into PSO algorithm. At the beginning of the generations, if the probability that $\left\{\vec{D}\right\}$ appears in one MOA_F exceeds a certain value ($T_{ep}$) in the current obtained solutions, then we will select solutions randomly on this dimension and make the $\vec{D}$ escape from current MOA_F to another one, thereby ensuring the uniformity of this dimension for the whole particle population.

In conclusion, the multi-objective scheduling optimization algorithm we proposed can offer users a solution to objectively evaluate the coverage quality under various monitoring requirements, efficiently schedule camera network, and further increase the utilization of camera monitoring resources.

## A controlled experiment with simulated data

In order to verify the rationality and validity of the proposed algorithm, we designed a controlled experiment with simulated data of camera network as well as directional road network, and compared initial monitoring solution with scheduling optimization solutions.

### Directional road network data

**Fig 2:**
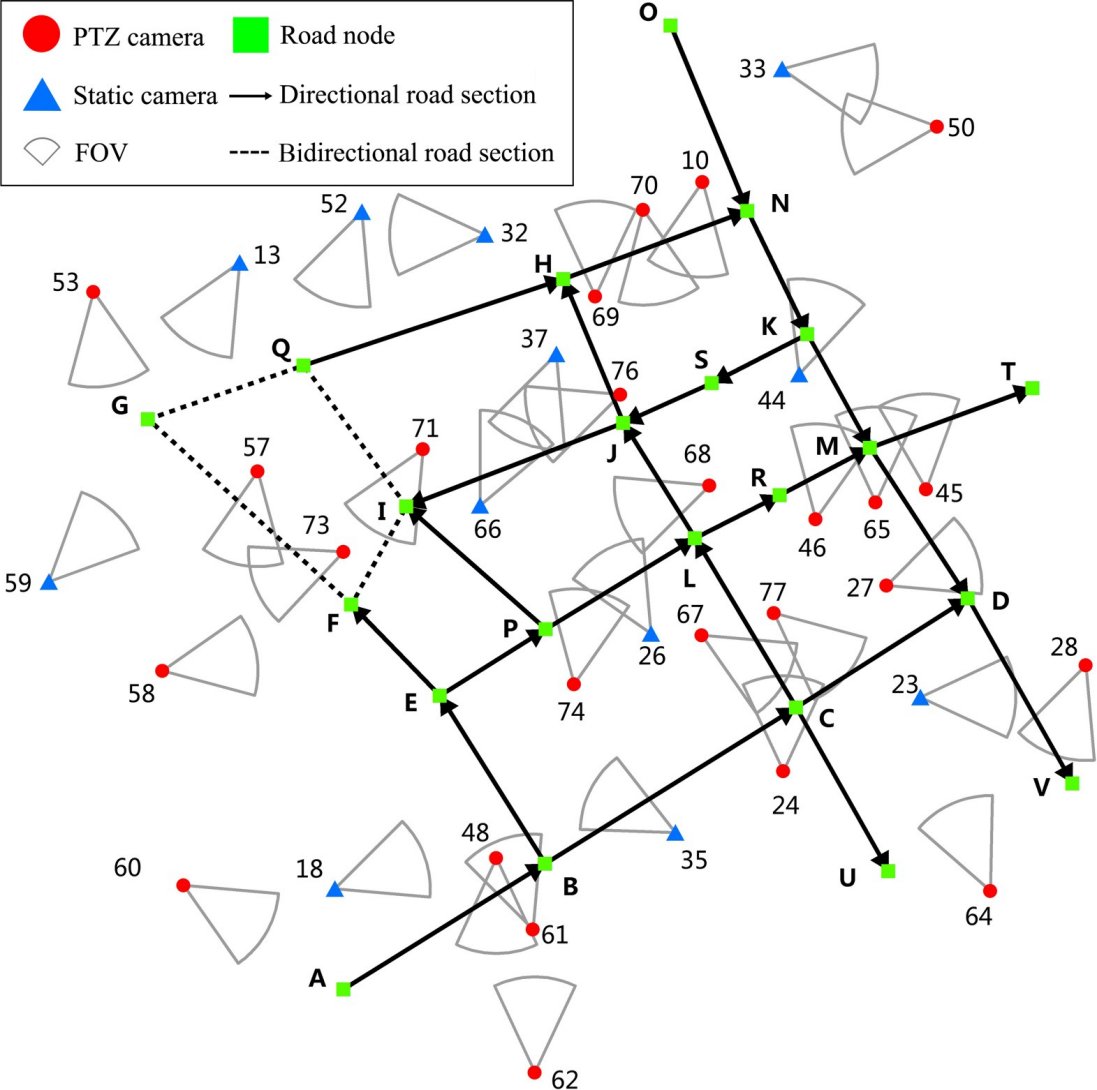
Initial camera and directional road network.

The green rectangle represents road node (similar to road node B), the solid line represents directional road section (similar to the directional road section A-B), and the dotted line represents bidirectional road section (similar to the bidirectional road section G-F). In this target road network, there are 29 road sections, of which 25 are directional and 4 are bidirectional (Fig 2).

### Camera network data

The blue triangle represents static camera, the red dot represents PTZ camera, and the gray sector represents the initial FOV. Therefore, the initial camera network has 37 cameras, of which 12 are static and 25 are PTZ (Fig 2). Furthermore, all the cameras are set in the same parameters (Table 2).

**Table 2:** Camera parameters.

Parameter	Value
**θ (°)**	50
**R (m)**	60
**TAng (°)**	135

In this road network, a single camera has three basic coverage types. The first only covers one road section (i.e., camera 35 only covers the road section B-C), the second covers several road sections at the same time (i.e., camera 68 covers both road sections P-L and C-L), and the third has several candidate road sections but only covers part of them at a time (i.e., camera 10 can cover road section O-N or H-N at a time, while camera 73 can cover road section F-I or G-F and E-F at a time). In this case, cameras may be independent of one another (i.e., cameras 35 and 24) or share same overlapping areas (i.e., cameras 48 and 61 have same coverage parts on road section A-B), Therefore, it is a fact that the coverage problem for a camera network is not the simple superposition of single cameras but generally exists the collaborative coverage among them, and it is necessary to schedule cameras for coverage optimization.

### Experiment set up

**Fig 3:**
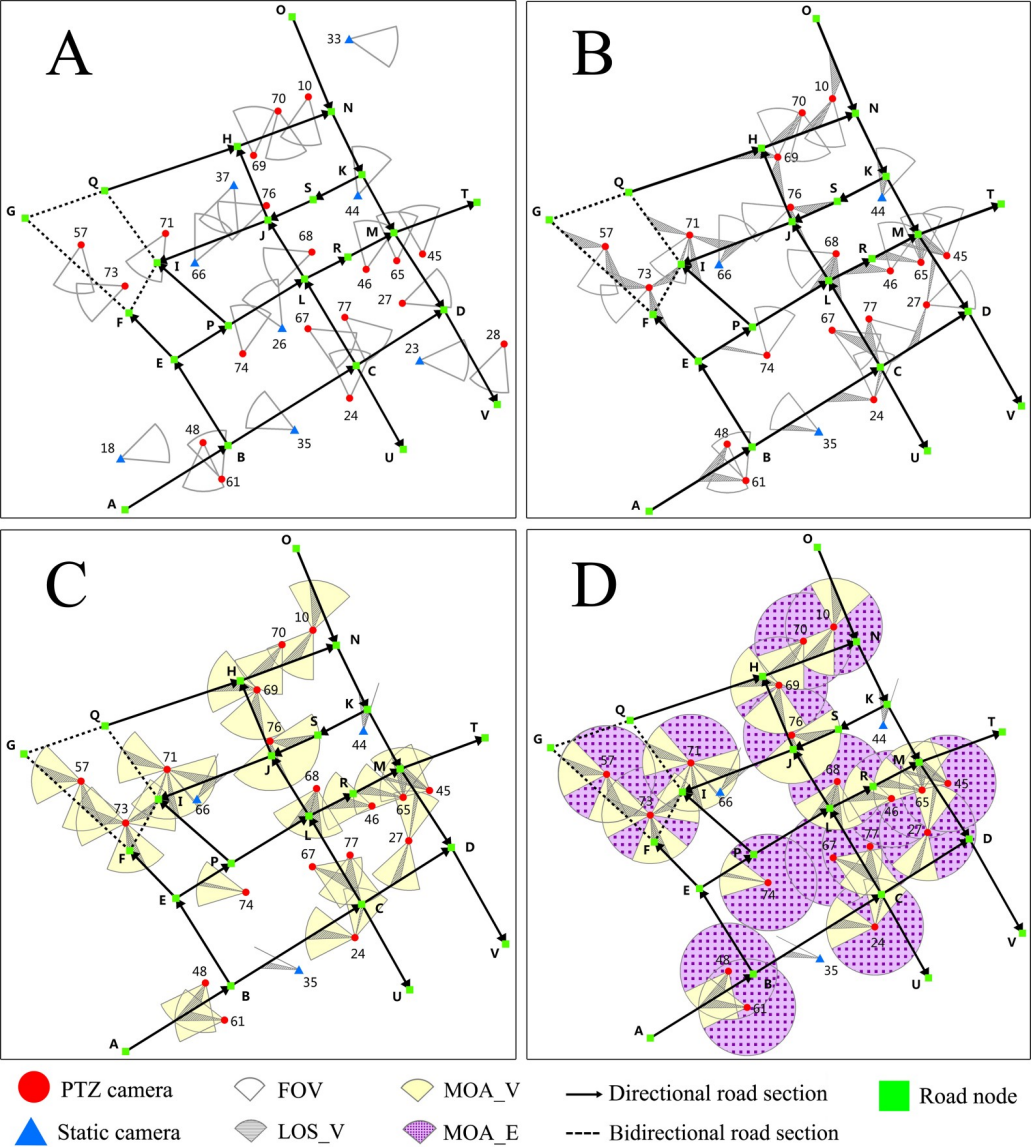
Experimental data. (A) Cameras after distance selection. (B) LOS_V and cameras after angle selection. (C) MOA_V. (D) MOA_E and MOA_F.

Firstly, we select cameras by distance and delete those that do not cover the target road network within R. We obtain 27 cameras, of which 8 are static and 19 are PTZ (Fig 3A). We then calculate the LOS_V of each camera with each road section. As shown in the shaded area (Fig 3B), the cameras without LOS_V have been deleted. In this way, we can obtain valid cameras that satisfy the constraints of distance and angle for scheduling. There are 21 cameras, of which 3 are static and 18 are PTZ (Fig 3B). According to the geometric relationship between $\vec{D}$ and θ, we can get MOA_V as represented by the yellow section (Fig 3C) and then calculate $k_{Ui}$, $k_{Li}$, $k_{c}$, and $k_{cn}$ as the important threshold values for *k* by Eqs (5)–(8). Table 3 presents these threshold values.

**Table 3:** Threshold values of expanding parameter of MOA.

Camera ID	MOA_F^a^	$k_{L}$	**$k_{U}$**	**$k_{c}$**	**$k_{cn}$**
**61**	244.26–308.11	2.32	2.32	2.32	2.80
**67**	79.14–146.63	2.17	2.17	2.17	
**68**	148.66–240.44	1.46	1.46	1.46	
**70**	179.68–251.76	2.00	2.00	2.00	
**74**	253.26–307.88	2.80	2.80	2.80	
**76**	57.15–227.41	0.57	0.57	0.57	
**77**	142.02–219.14	1.83	1.83	1.86	
**10**	179.68–248.10	0.91	0.57	0.91	
	331.08–407.51	0.57	0.91		
**24**	242.84–308.06	1.69	0.32	1.69	
	346.34–400.59	0.32	1.69		
**27**	167.60–227.71	1.14	1.00	1.14	
	341.98–396.92	1.00	1.14		
**57**	129.24–202.38	1.19	0.27	1.19	
	242.38–315.52	0.27	1.19		
**65**	249.53–312.22	1.51	0.05	1.51	
	319.07–369.92	0.05	1.51		
**69**	146.24–227.41	1.15	0.10	1.15	
	242.82–321.60	0.10	1.15		
**73**	29.09–99.27	0.5	0.22	0.50	
	130.01–202.38	0.22	0.27		
	242.38–316.56	0.27	0.50		
**71**	73.86–138.96	0.88	0.01	0.88	
	140.80–226.08	0.01	0.18		
	253.94–319.33	0.18	0.88		

^a^ The angle refers to the included angle between $\vec{D}$ and due north in clockwise direction.

We then set *k* = 2.8 and calculate MOA_E, which is represented by the purple section filled with dots (Fig 3D) and we can obtain the MOA_Fs of cameras that can participate in scheduling. The PSO algorithm parameters, such as population size, external archive size, and maximum number of generations has been set (Table 4).

**Table 4:** PSO algorithm parameters.

Parameter	Value	Explanation
**Population size**	100	Initialize the particle population
**External archive size**	500	Initialize the particle population
**Maximum number of generations**	500	Initialize the particle population
****ω****	0.4	Eq (10)
****$c_{1}$****	1	Eq (10)
****$c_{2}$****	1	Eq (10)
****$r_{1}$****	0.3	Eq (10)
****$r_{2}$****	0.4	Eq (10)
**$T_{ep}$**	70%	Perform escape

All the experiments are conducted using the environment with CPU of Intel(R) Core(TM) i5-6300HQ CPU @2.30GHz, and memory 4G. The program is developed with C++ on Microsoft Visual Studio 2012 (Table 5).

**Table 5:** Experimental environment.

Item	Configuration
**CPU**	Intel(R) Core(TM) i5-6300HQ CPU @2.30GHz
**Memory**	4.00G
**OS**	Windows 10 x64
**Development platform**	Microsoft Visual Studio 2012
**Development language**	C++

We eventually use the proposed multi-objective scheduling optimization algorithm to solve scheduling optimization set of camera network.

### Results

**Fig 4:**
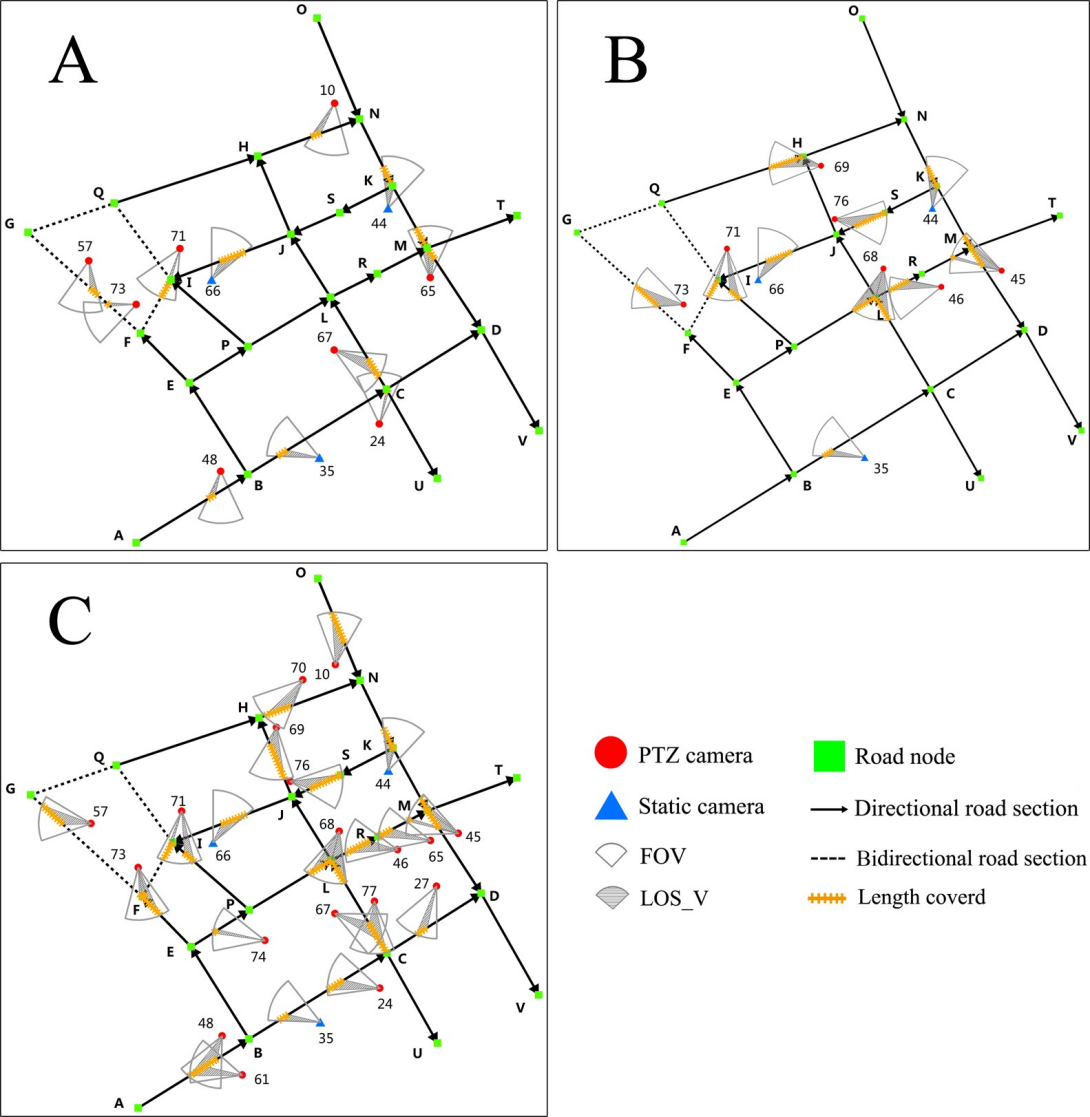
Evaluation of initial monitoring solution and scheduling optimization solutions. (A) Initial monitoring solution. (B) Scheduling optimization solution 1. (C) Scheduling optimization solution 2.

Two scheduling optimization solutions are used for quantitative analysis. The orange line represents the valid coverage length of road network (Fig 4A–4C).

We compared initial monitoring solution with two scheduling optimization solutions, and evaluated the optimization objectives mentioned above. Scheduling optimization solution 1 (Fig 4B) reduces the scheduling number of cameras by 1, increases the number of road sections covered by 4, and extends the length of road network covered by 153.67 meters or about 74.54% of the initial length covered (Table 6). It demonstrates the ability of the proposed method to balance multiple optimization objectives and obtain favorable optimization results.

**Table 6:** Comparison of initial monitoring solution and scheduling optimization solutions.

Optimization objective	Initial monitoring solution	Scheduling optimization solution 1		Scheduling optimization solution 2	
	Value	Value	Improvement	Value	Improvement
$Number_{\_road}(\{\vec{D}\})$	11	15	+4	21	+10
$Length_{\_road}(\{\vec{D}\})(m)$	206.17	359.84	+74.54%	619.24	+200.35%
$Number_{\_camera}(\{\vec{D}\})$	11	10	-1	21	+10

For scheduling optimization solution 2, all the valid cameras covering the target road network are participating in the scheduling (Fig 4C). The scheduling number of cameras increases by 10, the number of road sections covered increases by 10, and the length of road network covered increases by 413.07 meters or about 200.35% of the initial length covered (Table 6). It demonstrates that the proposed method can also take full advantage of available camera resources to maximize video monitoring quality.

In actual situations, users can set expanding parameter of MOA flexibly based on their preference and application requirements, and further strengthen the effectiveness and applicability of the method.

## Analysis and discussion

To evaluate effectiveness and robustness of the proposed method as well as the influence mechanism of expanding parameter of MOA on optimization results, three studies are carried out in this section. The first study compares all the obtained solutions with the true value solution set, while the second and third studies compare the optimization results among different groups of expanding parameter of MOA as well as camera parameters.

### Effectiveness

**Fig 5:**
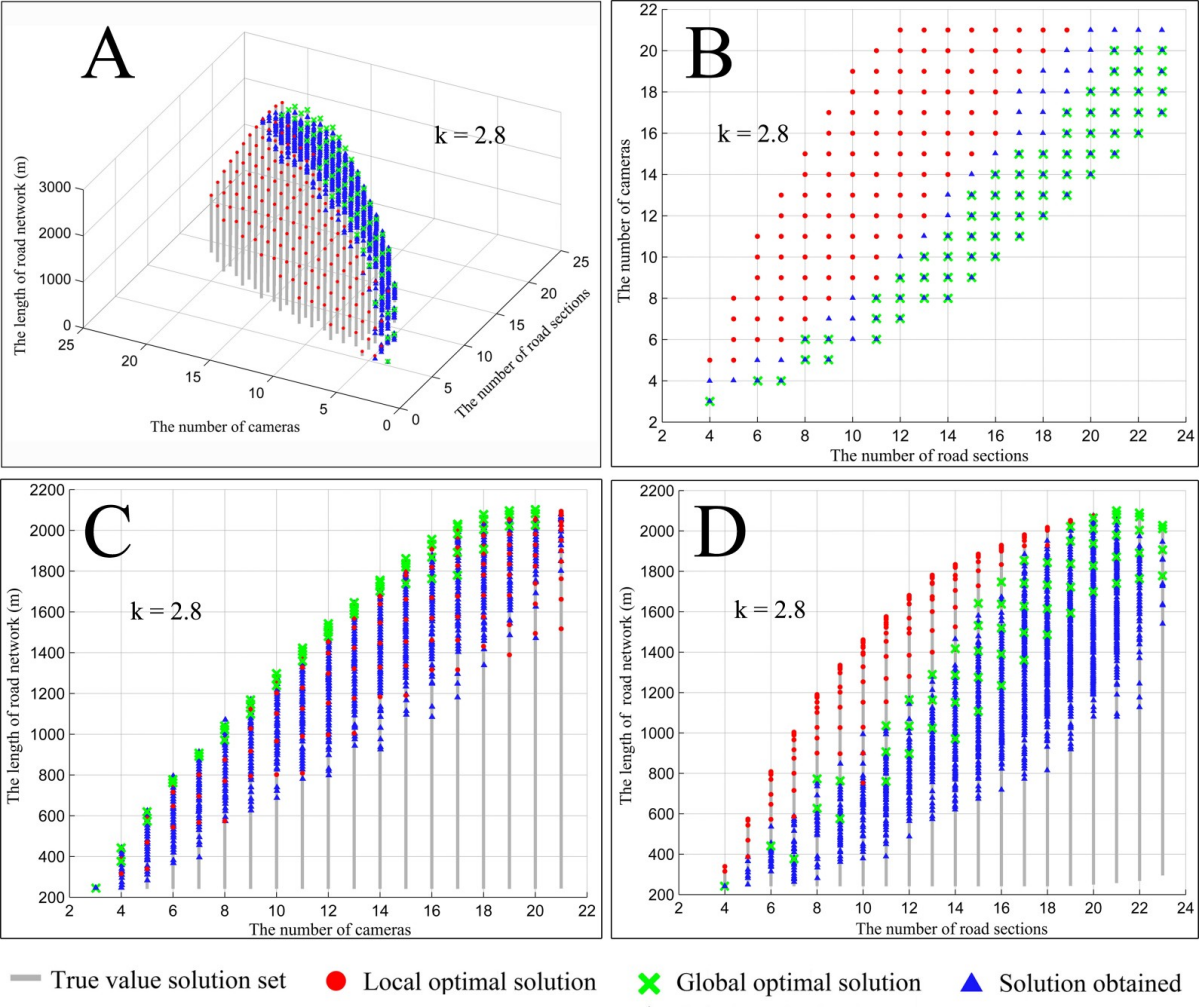
True value solution set and obtained solutions. (A) Scheduling number of cameras, number of road sections covered, and length of road network covered. (B) Number of road sections covered and scheduling number of cameras. (C) Scheduling number of cameras and length of road network covered. (D) Number of road sections covered and length of road network covered.

We exhaust all coverage types between camera network and road network as true value solution set with the precision of 1°. The x-axes represents the number of road sections, the y-axes represents the scheduling number of cameras, the z-axes represents the length of road network covered, and the gray segment represents the true value solution set (Fig 5A). Furthermore, each gray segment represents the scheduling solution set of different length of road network covered under a certain number of cameras and road sections. The red solid point, which is the top point of the gray segment, represents the local optimum solution, that is, the scheduling solution of the longest length covered under a certain number of cameras and road sections, and the green cross represents the global optimal solution or the best scheduling solution. The blue triangle represents the solutions obtained in 30 independent experiments using the parameters in Table 3.

From the distribution of the true value solution set (Fig 5B), we can find the dominant relationship of global optimal solutions among the local optimal solutions. Increasing the scheduling number of cameras can extend the longest length of road network covered to some extent. Due to the overlapping, after cameras reach a certain size, a further increase however, means only wasting monitoring recourse without further improvements (Fig 5C). Therefore, it is possible to use scheduling optimization methods to decrease the extra number of cameras without reducing monitoring quality and eventually increase resource utilization. Similarly, increasing the number of road sections covered can also extend the longest length of road network covered to some extent. However, given the conflict between different objectives, if we only focus on the number of road sections covered, a further increase will be at the expense of shortening the length of road network covered (Fig 5D). Therefore, only if the multiple optimization objectives are balanced, then we can obtain better scheduling optimization solutions of a camera network for road network coverage.

It is clear that the obtained optimization solutions are clustered around the local and global optimal solutions (Fig 5), which prove the proposed method can effectively coordinate and balance multiple optimization objectives, find optimization solutions under different coverage types, and ensure the diversity of optimization solution set.

### Influence of expanding parameter of MOA

In this section, we analyze the influence mechanism of expanding parameter of MOA on camera network scheduling in three aspects, summarize the general rules about how the parameter can dynamically control the optimization results, and provide some recommendations for its selection. Considering the range of the parameter and the distribution of its important threshold values (Table 3), we assign 0, 0.1, 0.32, 0.57, 1.0, 1.86, and 2.8 to *k* in the experiments, where 0 is the minimum value of *k* and 2.8 is equal to $k_{cn}$ and take the average of 30 independent experiments as the results for each group.

**Fig 6:**
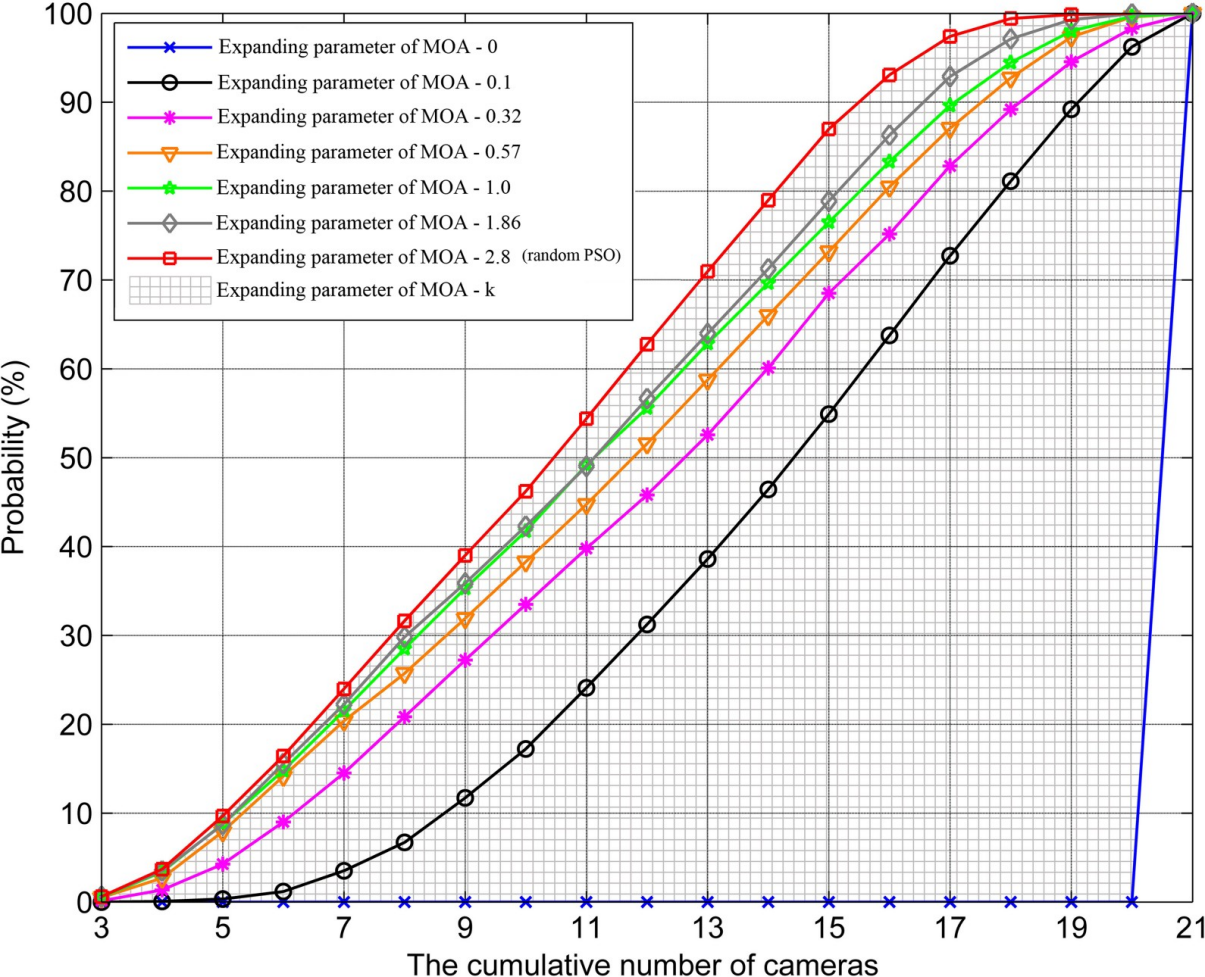
Influence of expanding parameter of MOA on optimization solution set distribution.

The x-axes represents all of the cameras number that no more than the value, and the y-axes represents the cumulative probability of all coverage types found by the proposed method (Fig 6). When *k* is equal to 0, the method can only find the coverage types of scheduling all the valid cameras. With the increasing of *k*, the probability of finding coverage types in a small number of cameras (<11) increases, while the probability in a large number of cameras (> = 11) decreases. Until *k* = $k_{cn}$, the probability in both large and small camera numbers is almost the same (46% and 54%, respectively), because the expanding parameter of MOA decides the range of MOA_E. The larger *k* corresponds to the larger MOA_E, the smaller probability for cameras to be selected to participate in the scheduling, and the larger probability for coverage types to be found in a small cameras number. Therefore, we can set the reasonable values of expanding parameter of MOA flexibly by calculating its important threshold values and distribution for more expected solutions.

**Fig 7:**
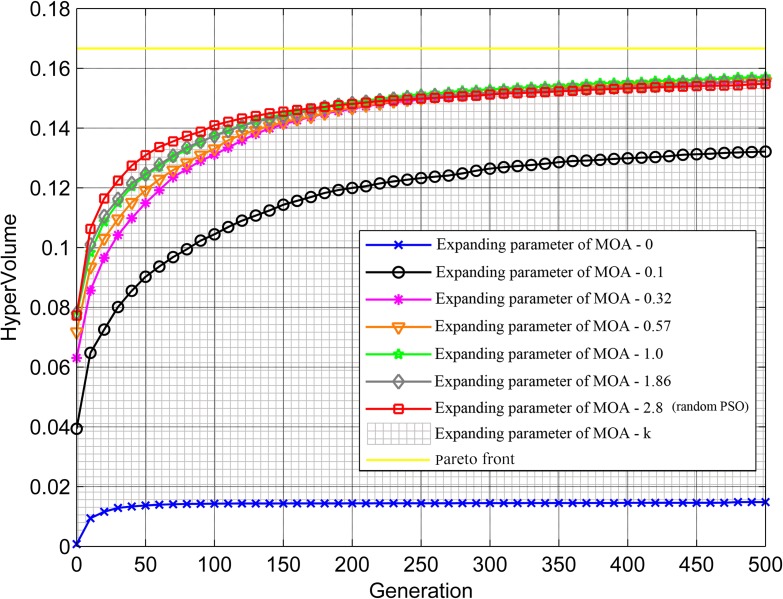
Influence of expanding parameter of MOA on optimization solution set performance.

Under different values of expanding parameter of MOA, the hypervolumes gradually all converge to the Pareto Front and the solution sets tend to stabilize as the number of generations increases (Fig 7). Moreover, the larger expanding parameter of MOA generates the larger hypervolume of initial particle population, because the parameter decides the spatial distribution of particle population at the beginning of generations, which, in turn, leads to the more uniform particle distribution, better diversity, and larger hypervolume. When the solution sets are convergent enough, the parameter corresponding to the maximum of hypervolume is equal to 1.0 instead of $k_{cn}$ (2.8), because a moderate value can favorably balance the coverage types in both large and small numbers of cameras and obtain better coordinated solution sets among convergence, uniformity, and universality.

**Fig 8:**
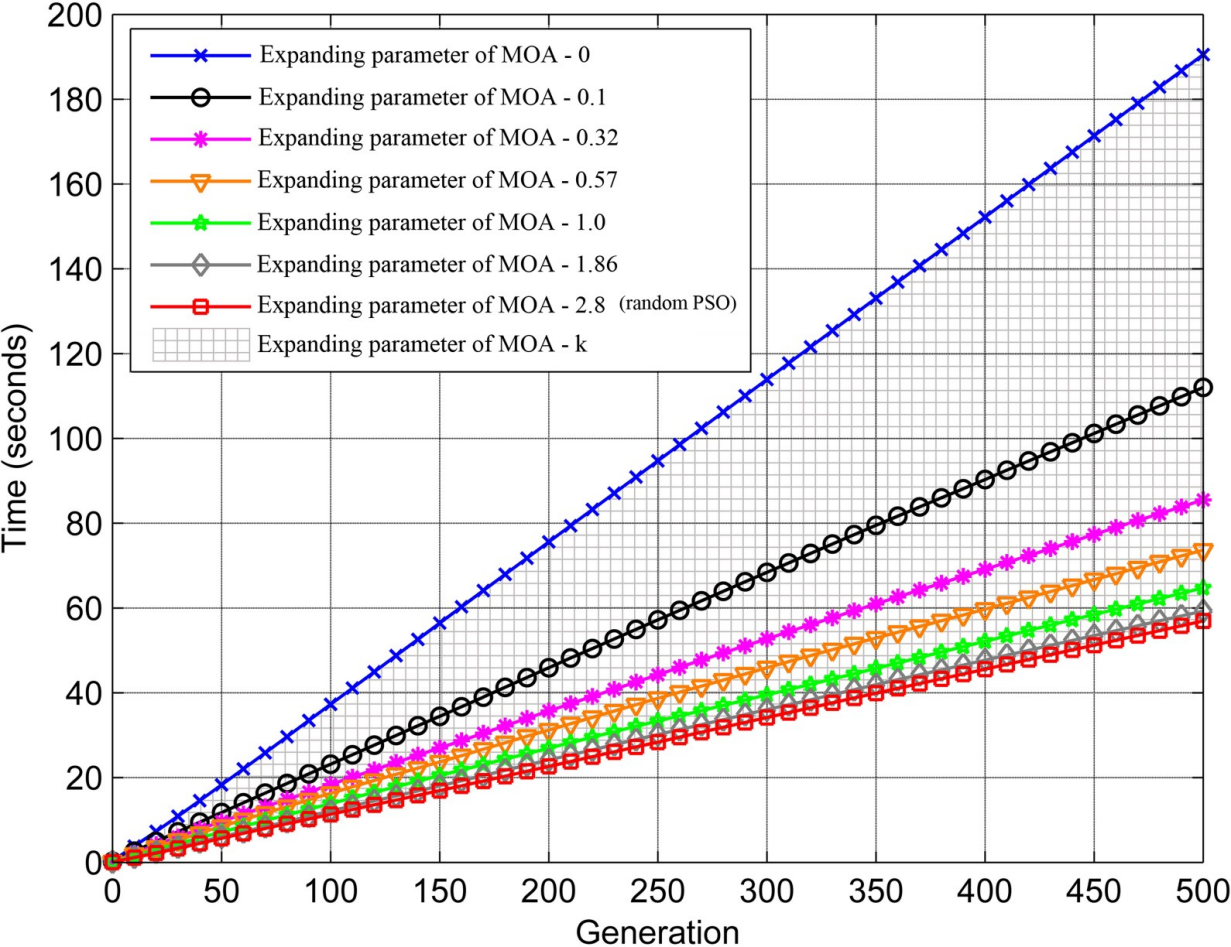
Influence of expanding parameter of MOA on optimization running time.

Under different values of expanding parameter of MOA, the running time of the proposed method linearly increases along with the generations (Fig 8). Specifically, the smaller expanding parameter of MOA requires the longer running time, because when the particles fly out of the feasible solution space during exploring, constraint handing is performed to change their flying direction and make them turn around. In this case, the smaller expanding parameter of MOA, the higher probability for particles to fly out, in turn, the more times of constraint handing performed, and the longer running time. Therefore, we can set proper number of generations in accordance with the requirements of practical applications or compare the hypervolume between adjacent generations in a certain step length until its rangeability is less than the threshold value, and then the solution set is convergent enough, we have obtained a favorable solution set without additional generations.

Furthermore, when *k* is equal to or greater than $k_{cn}$ (2.8), the proposed method will degenerate into the random PSO algorithm without constrains of expanding parameter of MOA. The shaded area represents the performance of our method (Figs 6–8), while the curve that *k* is equal to 2.8 represents the performance of the random PSO algorithm. It is clear that, in contrast, the proposed method presents a better flexibility among the performance, distribution, and running time of solution sets. In conclusion, the expanding parameter of MOA can enhance the applicability of PSO algorithm in practical monitoring tasks and support the scheduling optimization of camera on a road network.

### Influence of camera parameters

In this paper, camera parameters include θ, R, and TAng. To evaluate robustness of the proposed method, we design three groups of controlled experiments for each camera parameters separately, We set θ equals 30, 70, 90, R equals 45, 55, 65, TAng equals 90,120,150 (Table 7). According to the analysis presented in the previous section, our proposed method follows the same rules under different *k*. Therefore, all the controlled experiments are carried out under $k_{cn}$, and we take the average of 30 independent experiments as results.

**Table 7:** Camera parameters in experimental groups.

Camera parameter	Value 1	Value 2	Value 3
**θ (°)**	30	70	90
**R (m)**	45	55	65
**TAng (°)**	90	120	150

**Fig 9:**
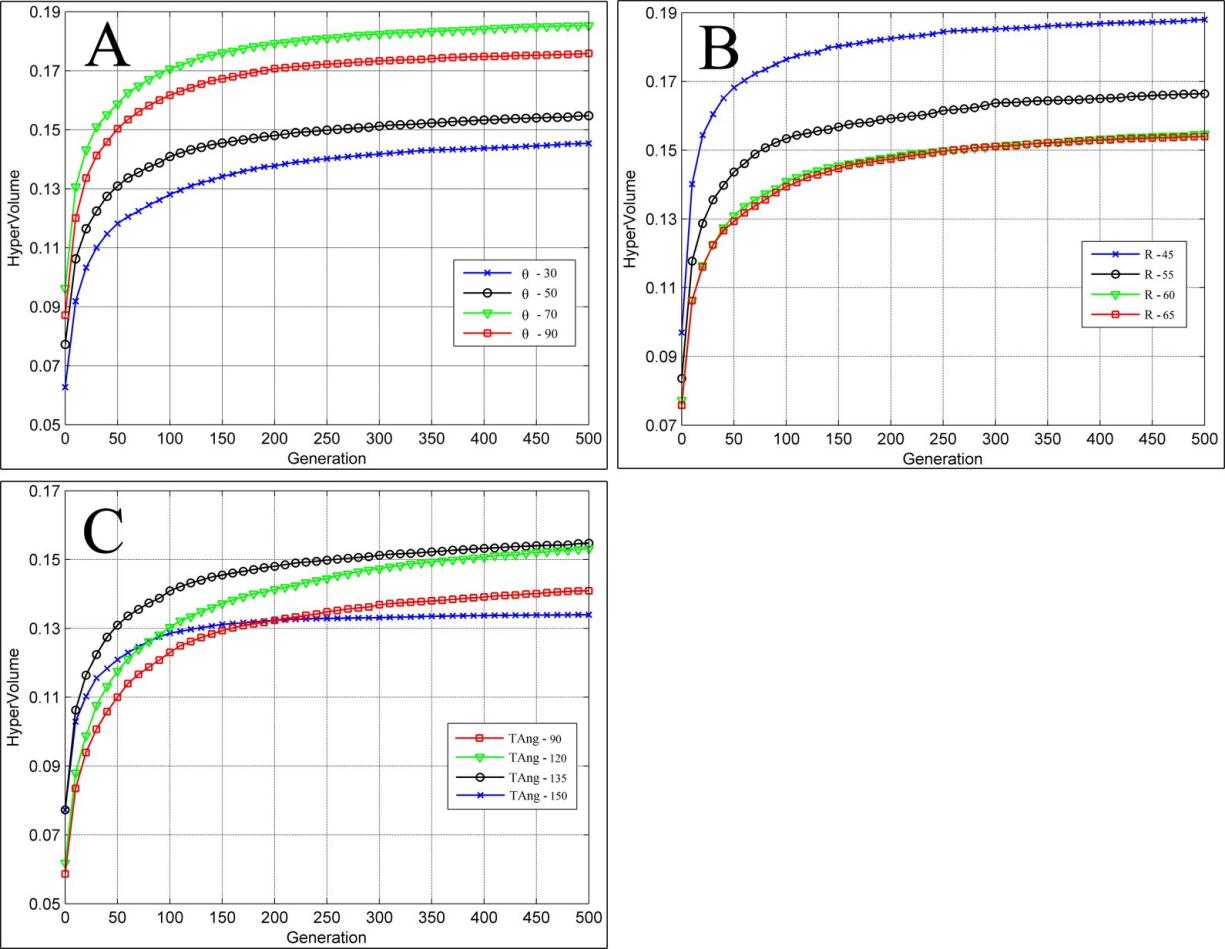
Influence of camera parameters on optimization results. (A) Experiments of θ. (B) Experiments of R. (C) Experiments of TAng.

Changing camera parameters can change the true value solution set. Therefore, after performing normalization under different systems of true value solution sets, hypervolumes of the solution sets become incomparable across different parameters, but still present a trend of convergence despite the changing of θ, R, and TAng (Fig 9).

Consequently, the proposed method is effective to obtain good scheduling optimization solutions and it can adapt to user application preference by changing the values of the expanding parameter of MOA for more expected solutions, and is robust to schedule and allocate monitoring resources in different scenarios.

## Conclusion

To address the optimization problem of camera and road network coverage, we propose a multi-objective scheduling optimization algorithm. We initially build a model for valid coverage of camera and directional road network, and then choose three of the most important aspects in general monitoring tasks as our optimization objectives, they are number of road sections covered, length of road network covered, and scheduling number of cameras. In this solution, we incorporate an expanding parameter of main optical axes into particle swarm optimization algorithm. Our new strategy divides the range of main optical axes of all the cameras to control the scheduling number, in turn, achieves a collaborative optimization of multiple objectives. On this basis, we analyze the influence of expanding parameter of MOA and camera parameters on the optimization results. The experiments demonstrate the effectiveness and robustness of the proposed method in solving complex optimization problem of camera and road network coverage, and it can flexibly coordinate and schedule cameras, maximize the utilization of these resources. In actual monitoring tasks, this method is not only confined to the three aforementioned optimization objectives. If necessary, other optimization objectives can be directly input into the model without changing the algorithm process, due to the unified optimization strategy for different optimization objectives. This method can also provide some recommendations for other directional sensor resources in multi-objective optimization allocation.

## Supporting information

S1 File CameraFile. The camera data in this study.
(TXT)

S2 File RoadEdgeFile. The road section data in this study.
(TXT)

S3 File RoadPointFile. The road node data in this study.
(TXT)

S4 File SourceCode. The source code of the proposed method in this paper and it is developed by C++.
(ZIP)
